# Supplementary material for: Atomic-scale combination of germanium-zinc nanofibers for structural and electrochemical evolution
Source: Nat Commun. 2019 May 30;10:2364. doi: 10.1038/s41467-019-10305-x (PMC6542799; doi:10.1038/s41467-019-10305-x)
Supplement: Supplementary file 1 — Supplementary Information [file 41467_2019_10305_MOESM1_ESM.pdf]

Supplementary Information

Atomic-scale combination of germanium-zinc nanofibers for structural and electrochemical evolution

Song et al.

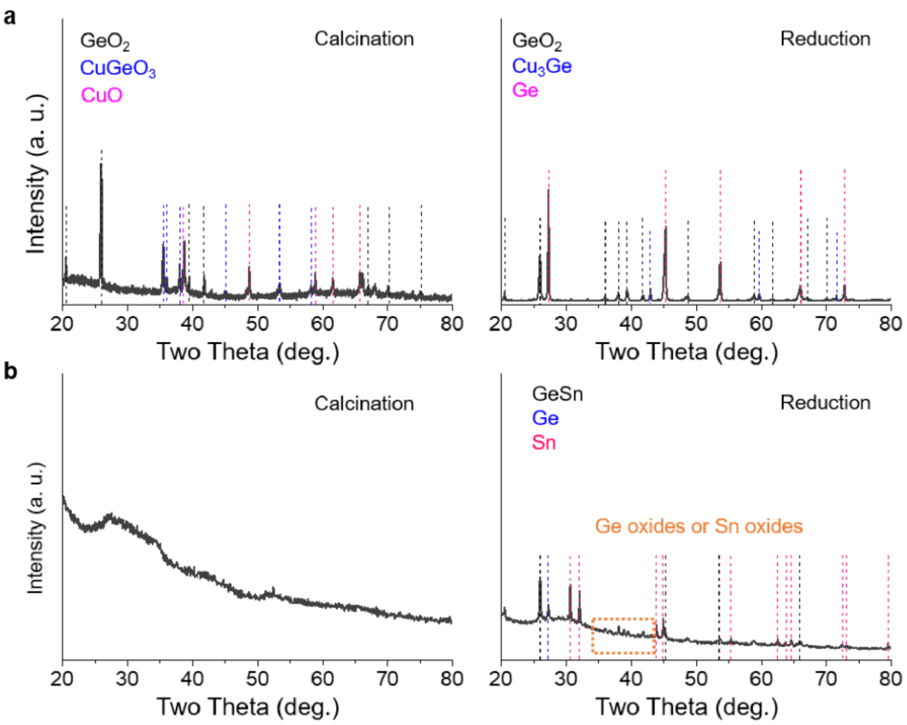

Supplementary Figure 1. Microstructure of Ge-Cu and Ge-Sn NFs. XRD patterns of **a** Ge-Cu and **b** Ge-Sn composite NFs after calcination and reduction process.

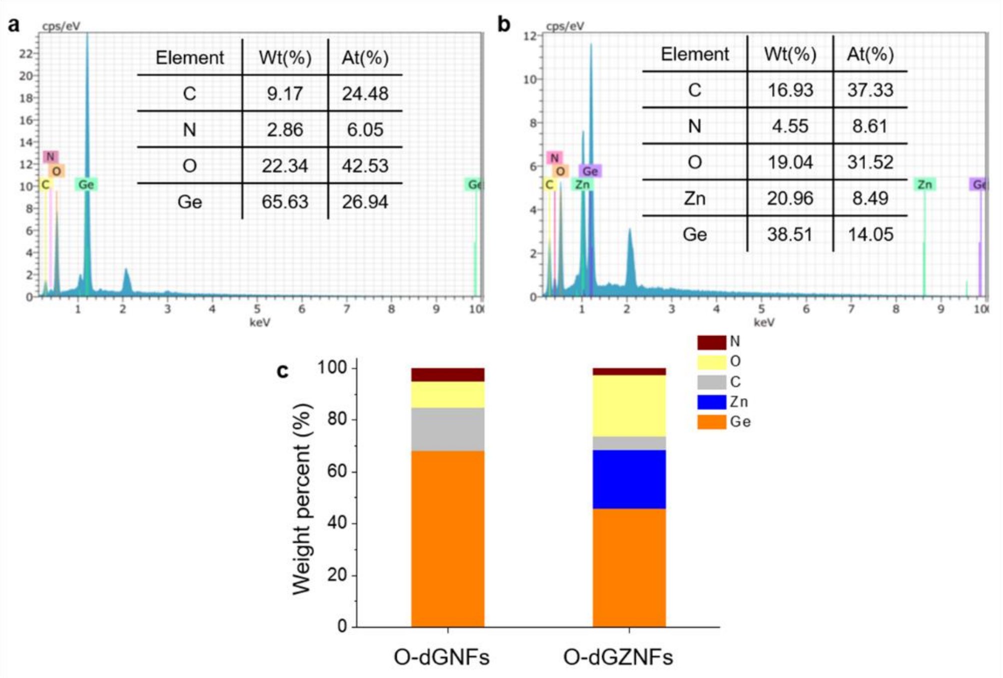

Supplementary Figure 2. Elemental characterization of each nanofiber. EDS spectra results from SEM measurement of **a** O-iGNFs and **b** O-iGZNFs. **c** EA/ICP summary of O-dGNFs and O-GZNFs.

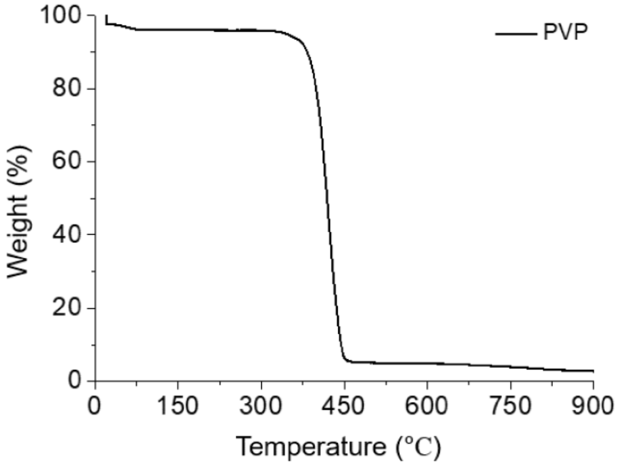

Supplementary Figure 3. Decomposition behavior of PVP. Thermogravimetric analysis (TGA) of pure PVP polymer in air.

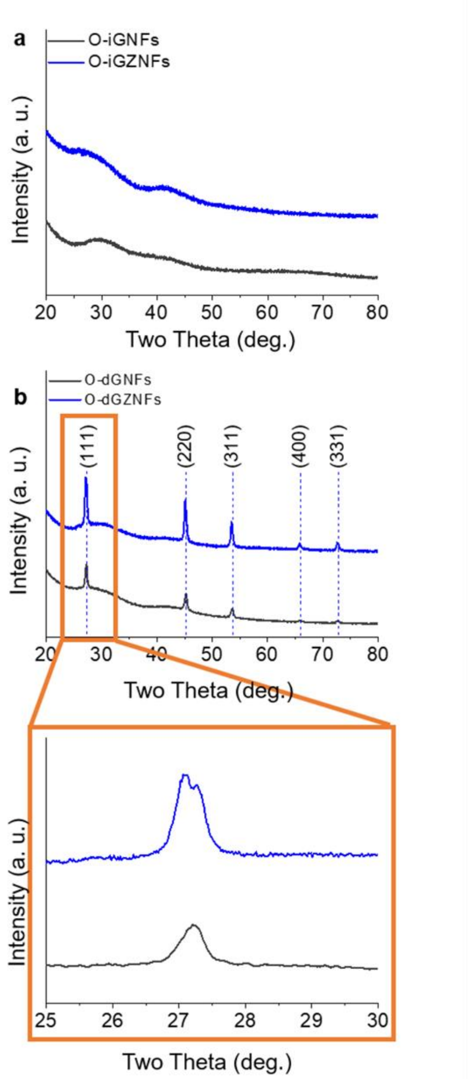

Supplementary Figure 4. Microstructure of Ge-Zn nanofibers. XRD patterns of **a** O-iGNFs, O-iGZNFs before reduction reaction. All components exist amorphous phases where broad peaks indicate amorphous carbon in NFs and **b** O-dGNFs, O-dGZNFs after reduction reaction. Peaks of O-dGZNFs are slightly shifted and partially separated due to the distorted Ge d-spacing.

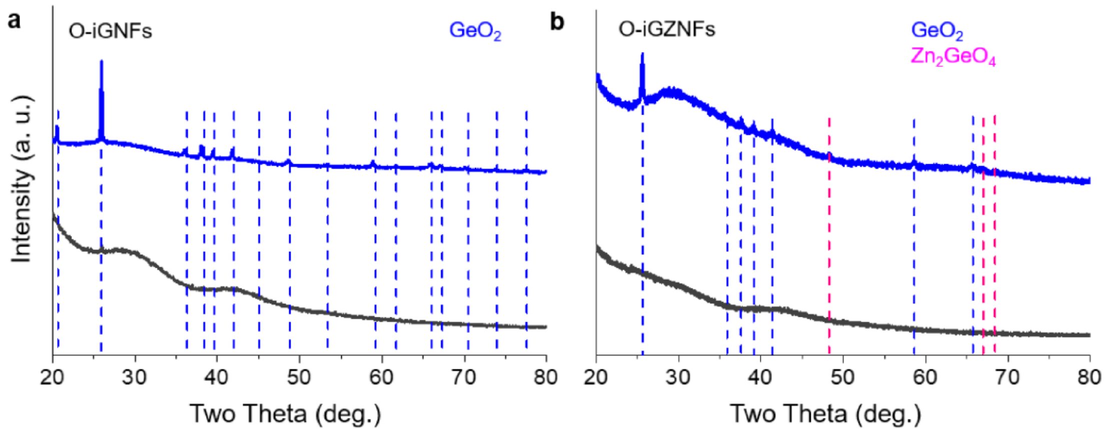

Supplementary Figure 5. Microstructure of Ge-Zn nanofiber for calcination temperature modification. XRD patterns of **a** O-iGNFs and **b** O-iGZNFs calcined at 600℃ (grey) and 650℃ (blue) in air.

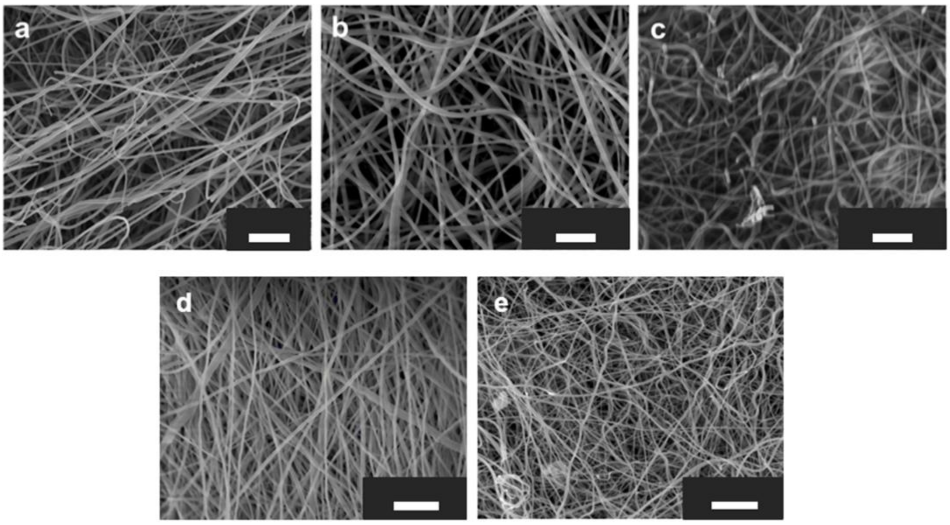

Supplementary Figure 6. Surface morphologies of nanofibers in each step. SEM images of **a** as-spun, **b** as-calcined, **c** as-reduced sample of O-dGNFs series, **d** as-calcined, and **e** as-reduced sample of O-dGZNFs series. Scale bars: **a** 1 μm, **b,c** 500 nm, and **d,e** 1 μm.

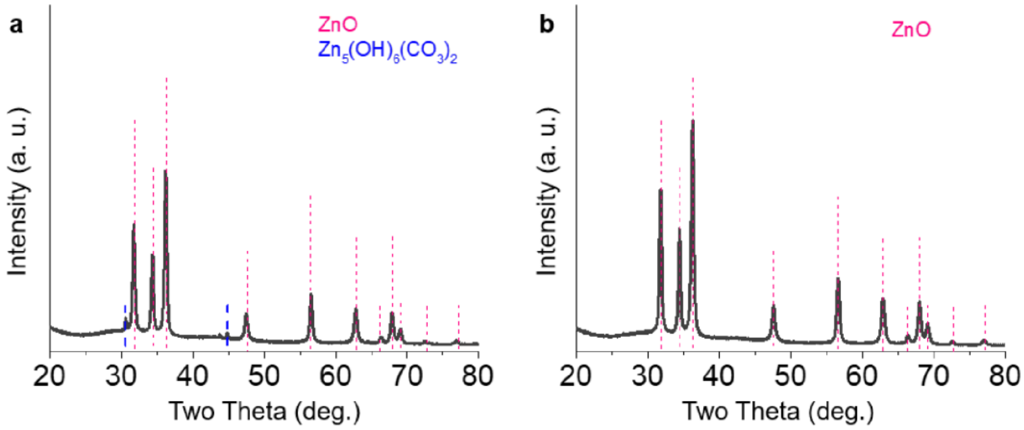

Supplementary Figure 7. Microstructure of pure ZnO nanofibers in each step. XRD patterns of **a** as-calcined and **b** as-reduced ZnO nanofibers. As-synthesized pure ZnO NFs cannot be reduced into Zn metal. It means the activation energy of breaking Zn-O bonds is not enough at given reduction temperature (600° C).

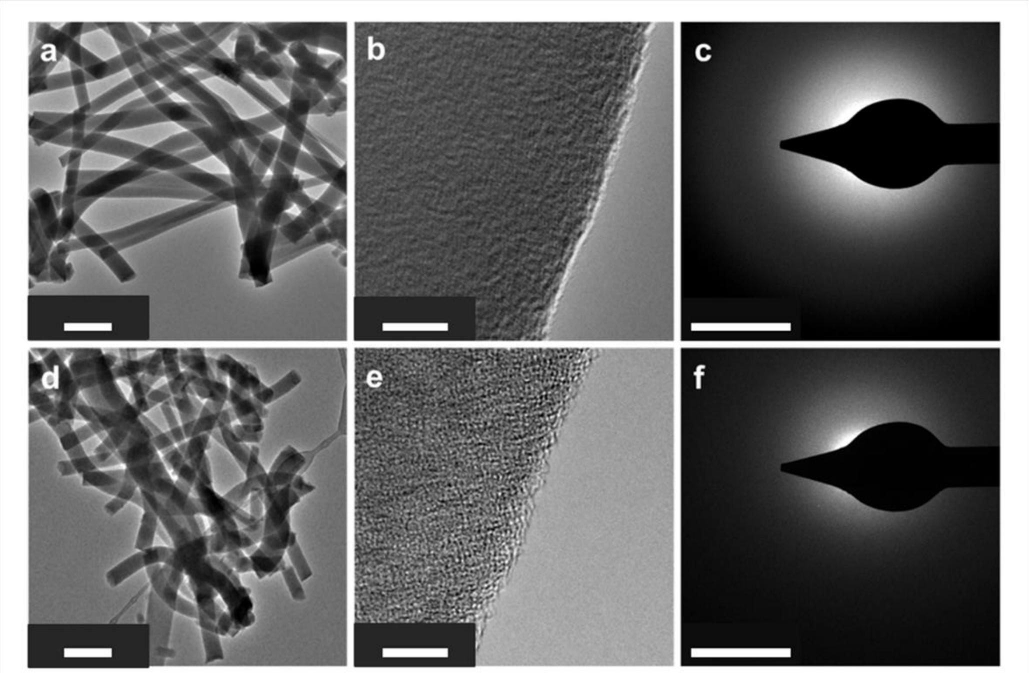

Supplementary Figure 8. Crystalline structure of O-iGNFs and O-dGNFs. TEM images and SAED patterns of **a-c** O-iGNFs and **d-f** O-dGNFs. As-calcined nanofibers, O-GNFs, shows amorphous SAED patterns because GeO_2 existed in amorphous state. On the other hand, although O-dGNFs have some crystalline lattice based on XRD patterns, they barely exhibit lattice fringe and crystal spot because many portions are composed to GeO_2 and carbon matrix due to Ge sublimation on reduction process. Scale bars: **a,d** 200 nm, **b,e** 5 nm, and **c,f** 5 1/nm.

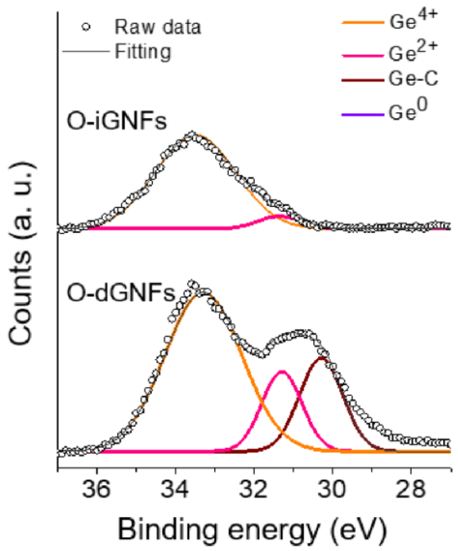

Supplementary Figure 9. Ge oxidation states of O-iGNFs and O-dGNFs. Core level XPS spectrum of O-iGNFs and O-dGNFs. Ge^{2+} still remains in O-dGNFs due to the existence of large portions of GeO_2. The sublimation of Ge brings out this result while reducing Ge^0 peak at nanofiber surface.

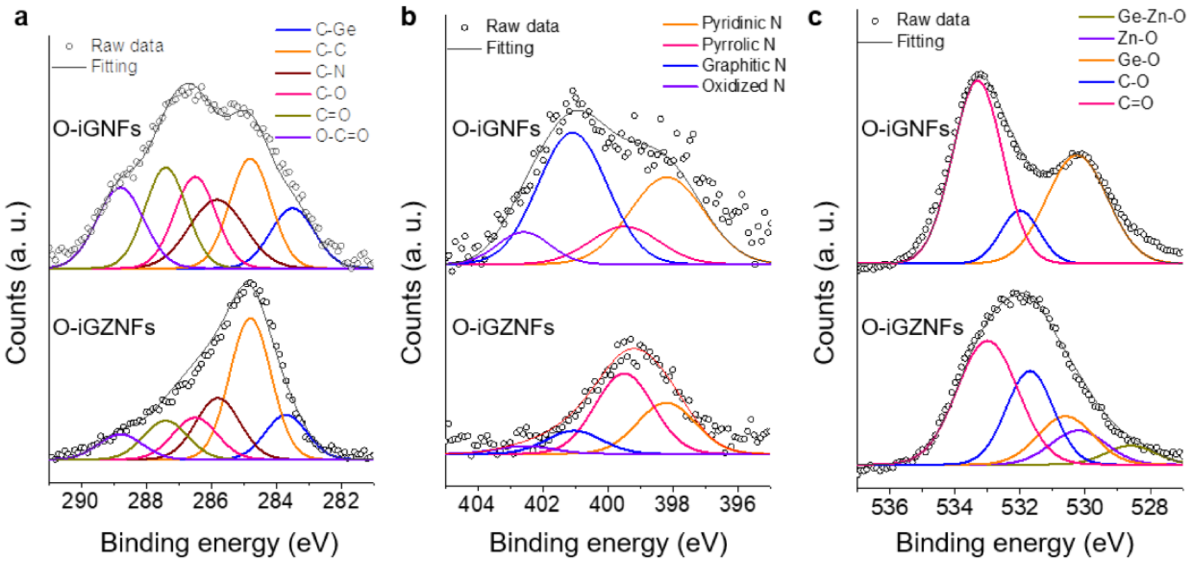

Supplementary Figure 10. Binding configuration of O-iGNFs and O-iGZNFs. Core level XPS spectra of **a** C 1s, **b** N 1s, and **c** O 1s of O-iGNFs and O-iGZNFs.

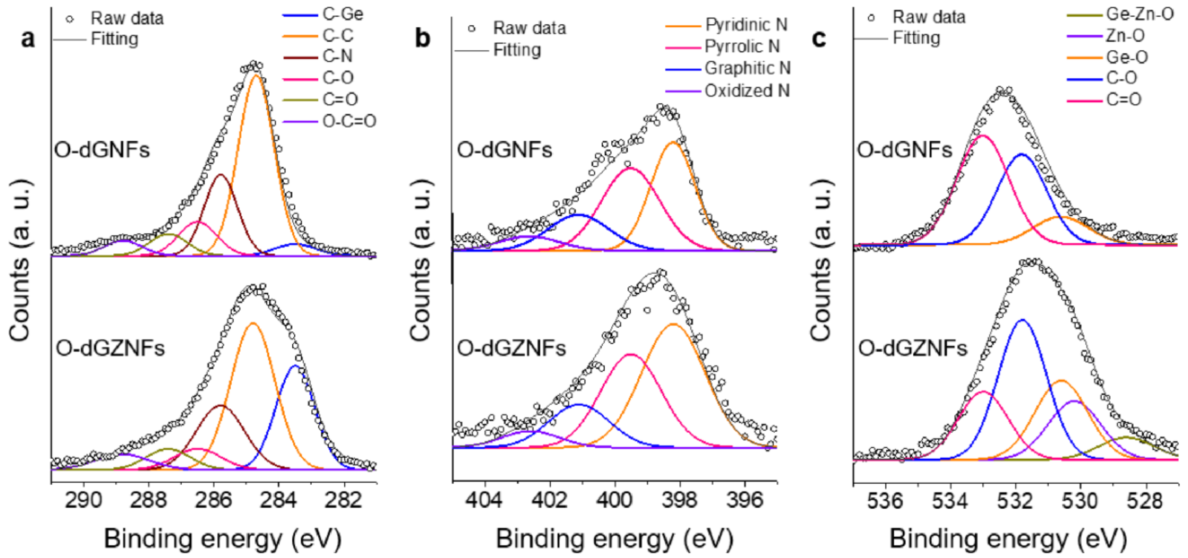

Supplementary Figure 11. Binding configuration of O-dGNFs and O-dGZNFs. Core level XPS spectra of **a** C 1s, **b** N 1s, and **c** O 1s of O-dGNFs and O-dGZNFs.

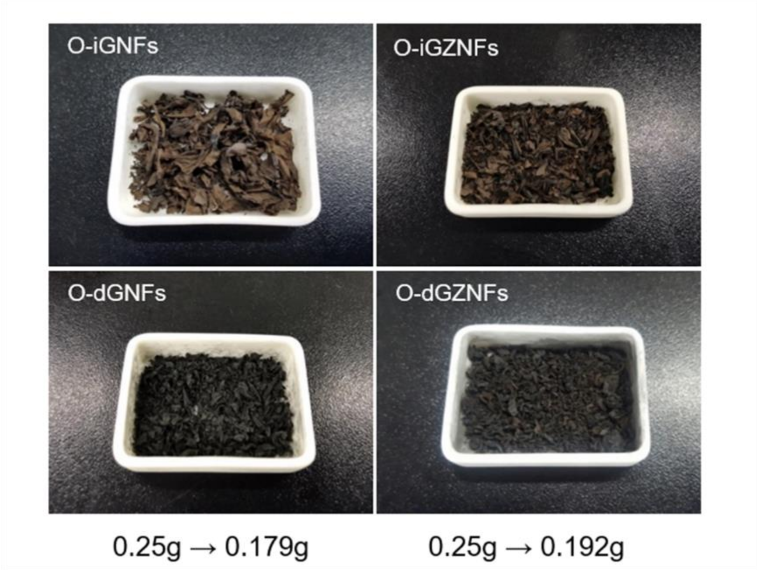

Supplementary Figure 12. Optical images of each nanofiber formed by reduction process. Photographs to confirm weight change by reduction process.

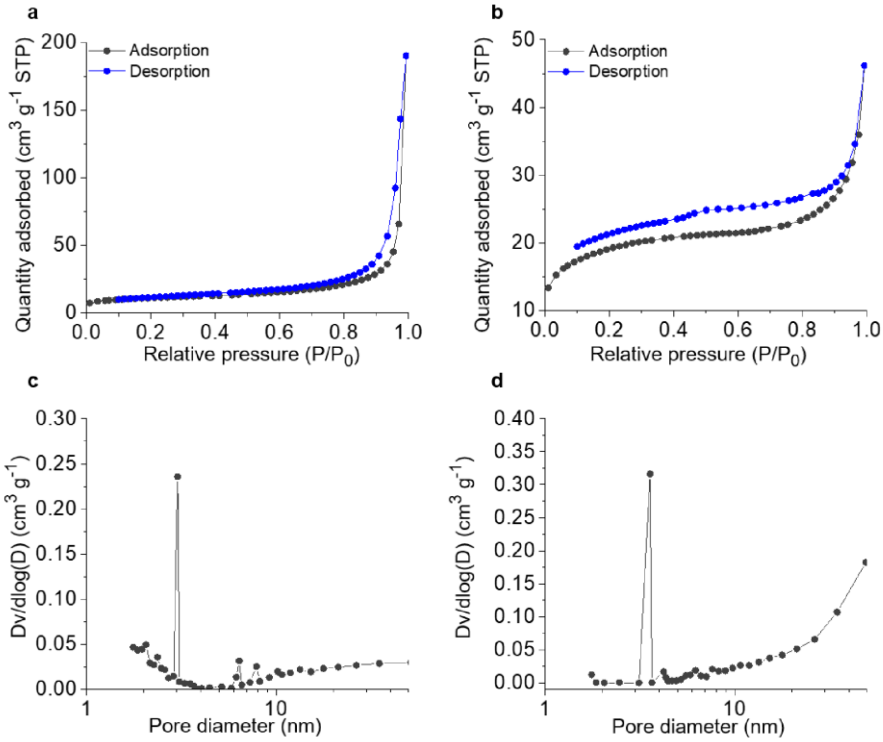

Supplementary Figure 13. Surface and pore characterization of as-reduced nanofibers. Isotherm plots of N_2 adsorption/desorption of a O-dGNFs and b O-dGZNFs with BET surfaces; 37.1 m^2 g^{-1} and 61.3 m^2 g^{-1}. Pore size distribution of c O-dGNFs and d O-dGZNFs.

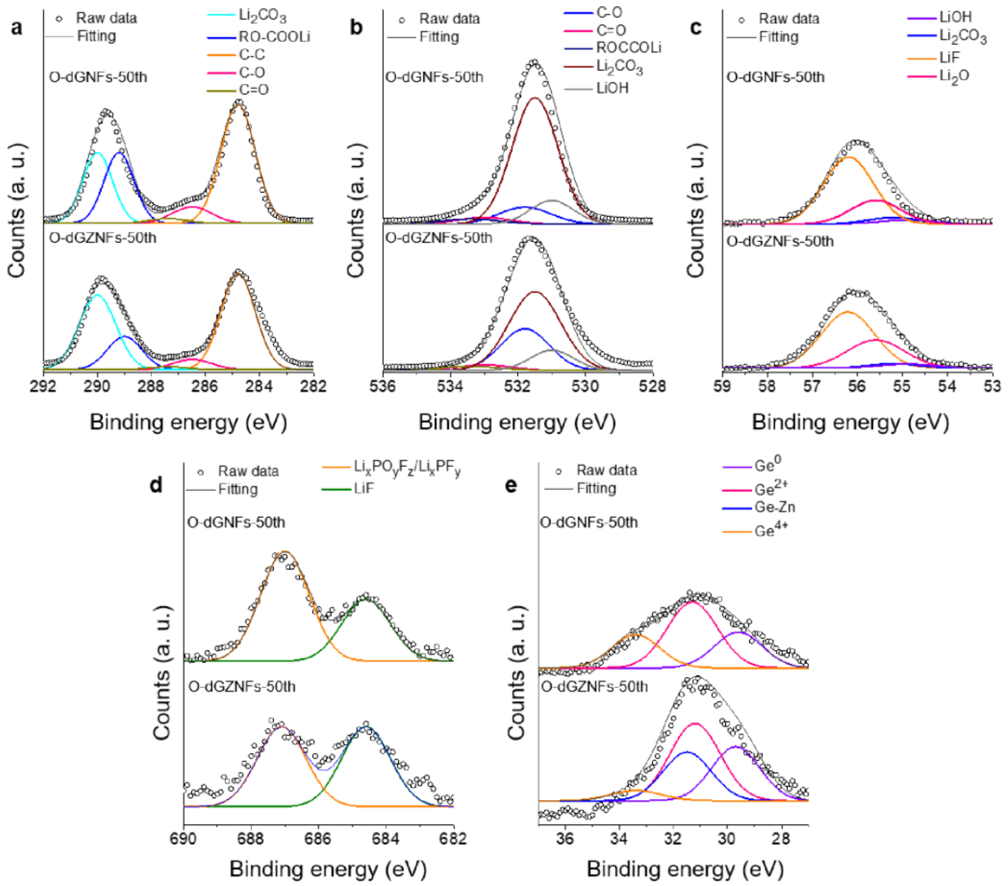

Supplementary Figure 14. Binding configuration of O-dGNFs and O-dGZNFs after 50 cycles. Core level XPS spectra of **a** C 1s, **b** O 1s, **c** Li 1s, **d** F 1s, **e** Ge 3d of O-dGNFs-50th and O-dGZNFs-50th after slightly etching the surface of electrodes to remove SEI layer.

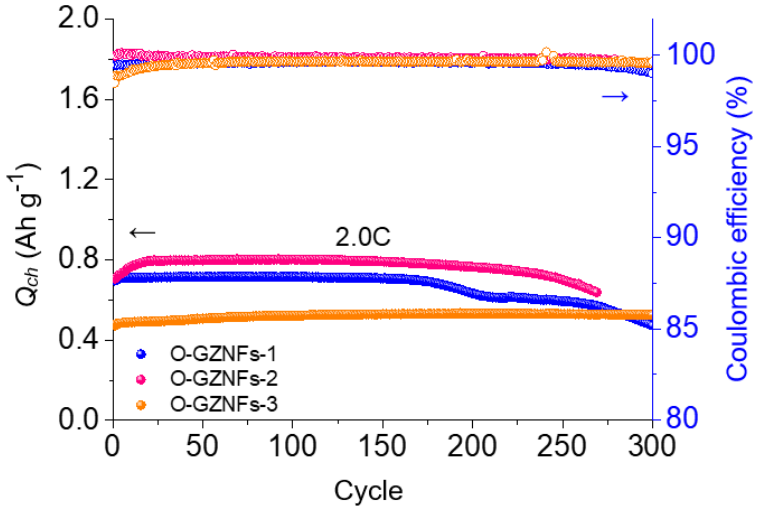

Supplementary Figure 15. Capacity retention of O-GZNFs with different oxygen contents in nanofibers. Charge capacity retention of O-dGZNFs-1, 2, and 3 depending on different oxygen ratios (O-dGZNFs-1: Ge/Zn=10/1, O-dGZNFs-2: Ge/Zn=5/1, and O-dGZNFs-3:Ge/Zn=1/1).

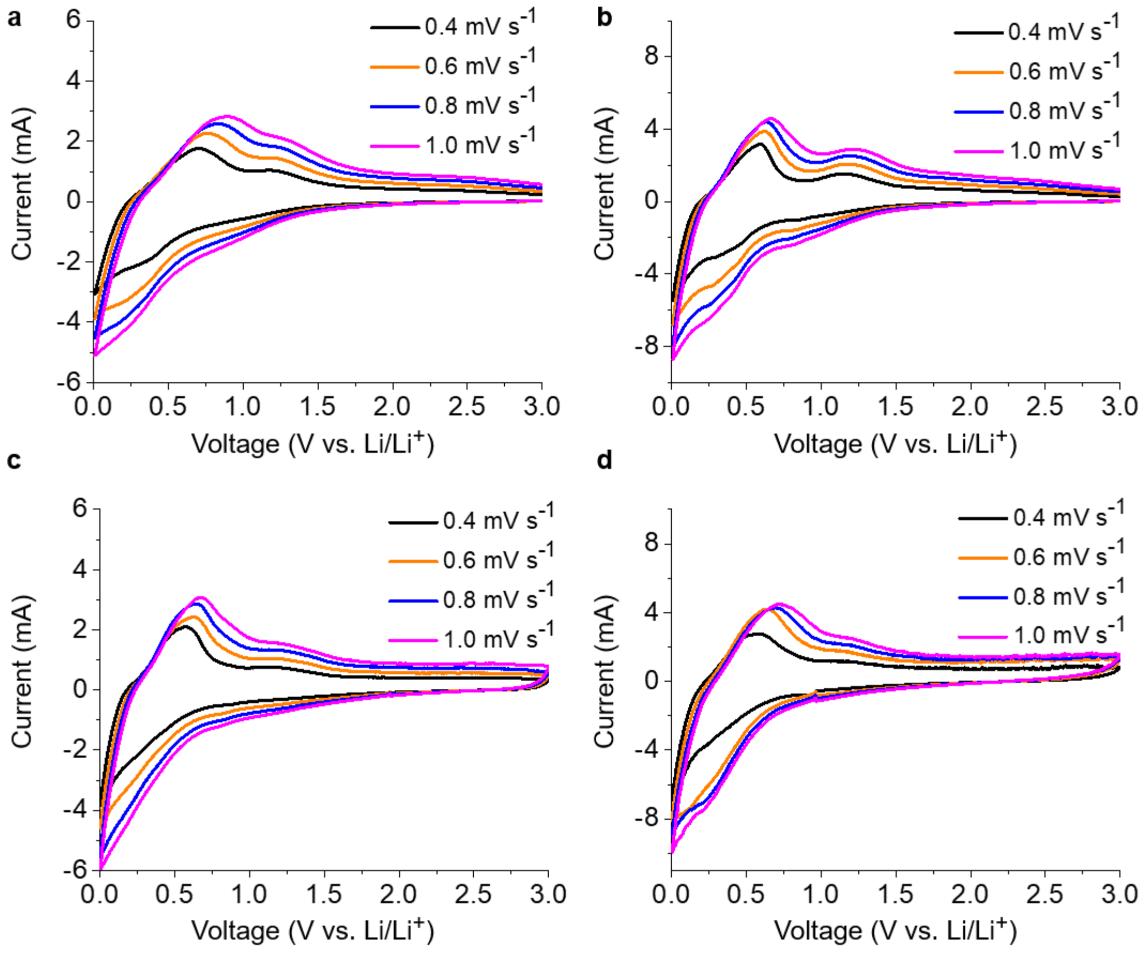

Supplementary Figure 16. Cyclic voltammetry measurement at various scan rates. **a** O-dGNFs, **b** O-dGZNFs, **c** O-dGNFs-50th, and **d** O-dGZNFs-50th.

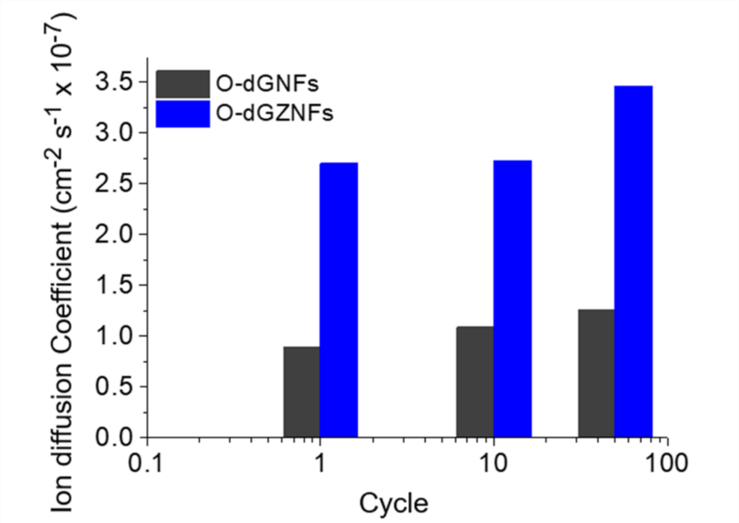

Supplementary Figure 17. Comparison of Li-ion diffusion coefficients for O-dGNFs and O-dGZNFs depending on cycles.

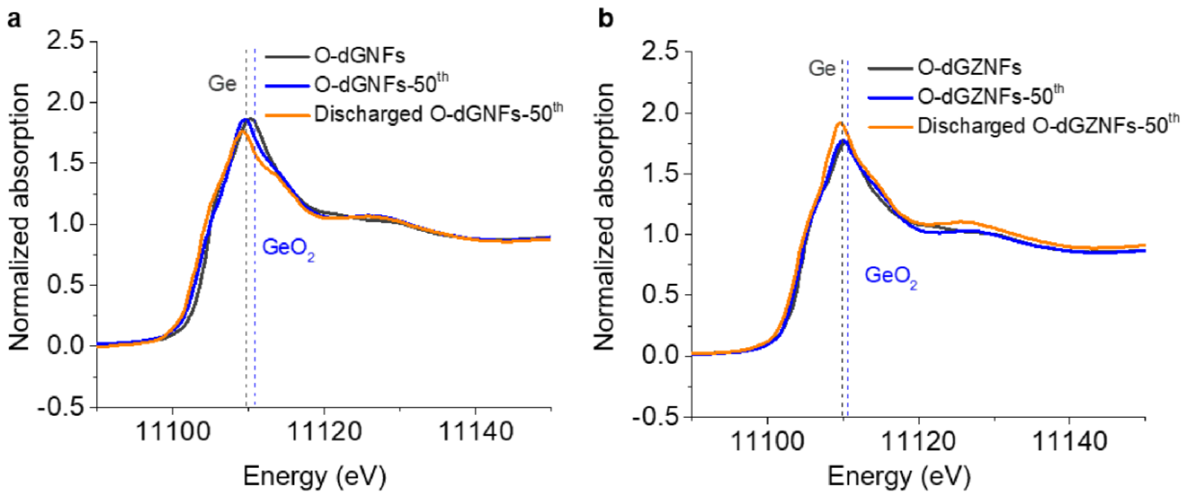

Supplementary Figure 18. Oxidation states of O-dGNFs and O-dGZNFs. XANES spectra of **a** O-dGNFs and **b** O-dGZNFs at various states related to **Fig. 4c, d.**

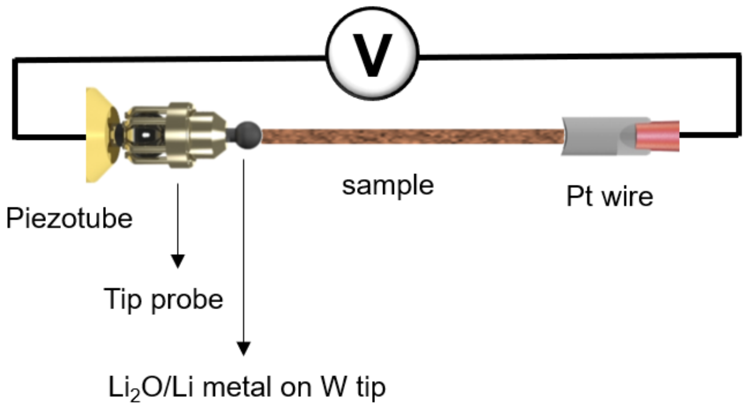

Supplementary Figure 19. Schematic illustration of *in situ* nanobattery STM holder.

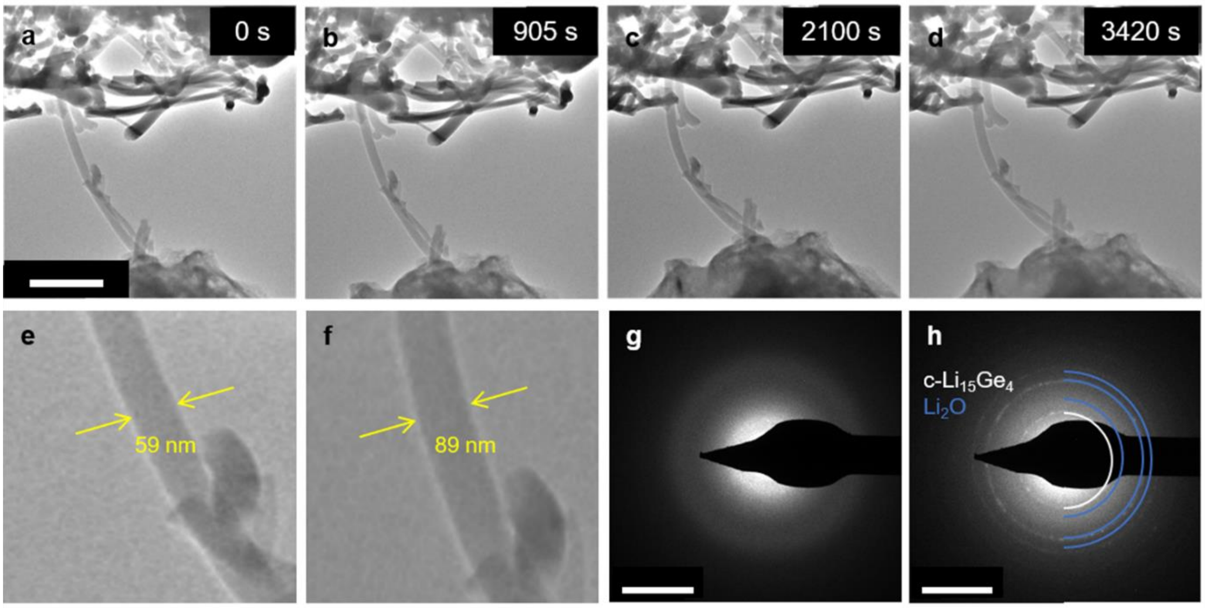

Supplementary Figure 20. *In situ* monitoring of O-dGNFs morphology during lithiation. Time-resolved TEM images on lithiation process of **a-d** O-dGNFs. Magnified TEM iamges correspond to **e** pristine and **f** fully lithiated sample. SAED patterns of **g** pristine and **h** fully lithitated sample are consistent with **e** and **f**, respectively. Scale bars: **a-d** 500 nm and **g,h** 5/nm.

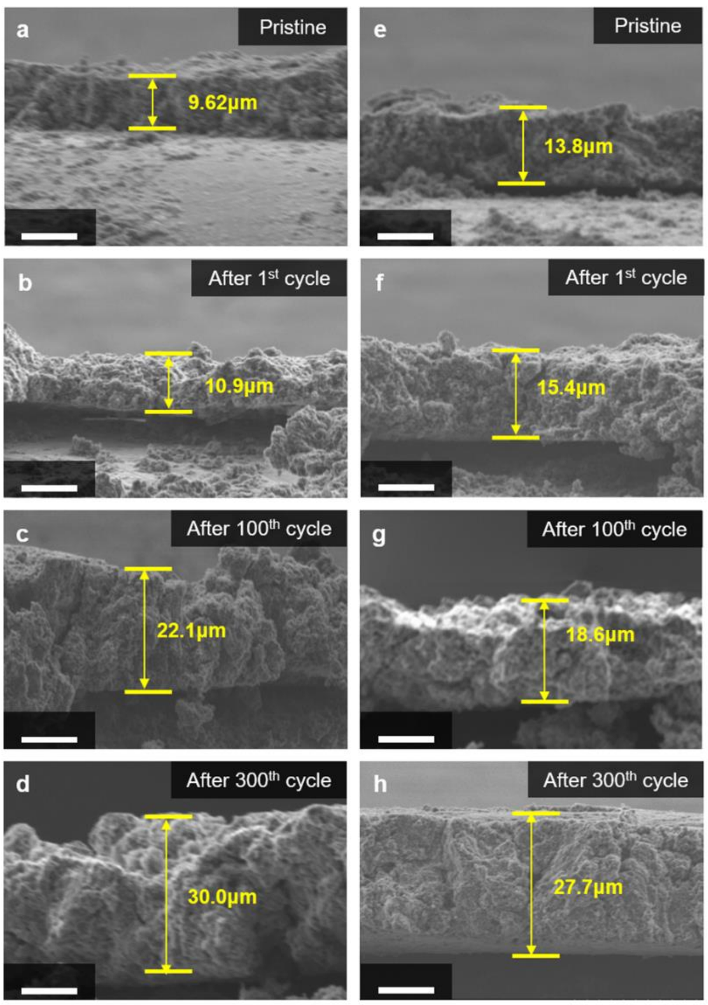

Supplementary Figure 21. Electrode swelling results of O-dGNFs and O-dGZNFs at featured cycles. Electrodes swelling results of **a-d** O-dGNFs and **e-h** O-dGZNFs for pristine electrode, after 1st cycle, after 100th cycle, and after 300th cycle. Scale bar: **a-h** 10 μm

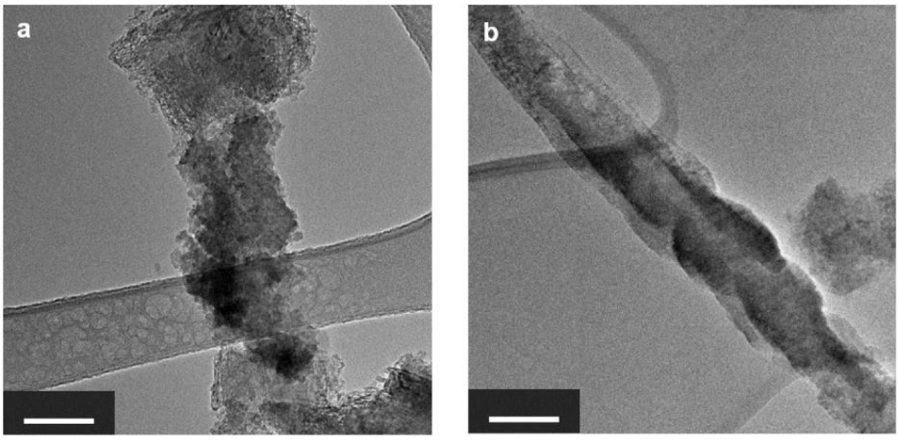

Supplementary Figure 22. Morphology change of O-dGNFs and O-dGZNFs after 50 cycles. TEM images for **a** O-dGNFs-50th and **b** O-dGZNFs-50th. Scale bar: **a,b** 100 nm.

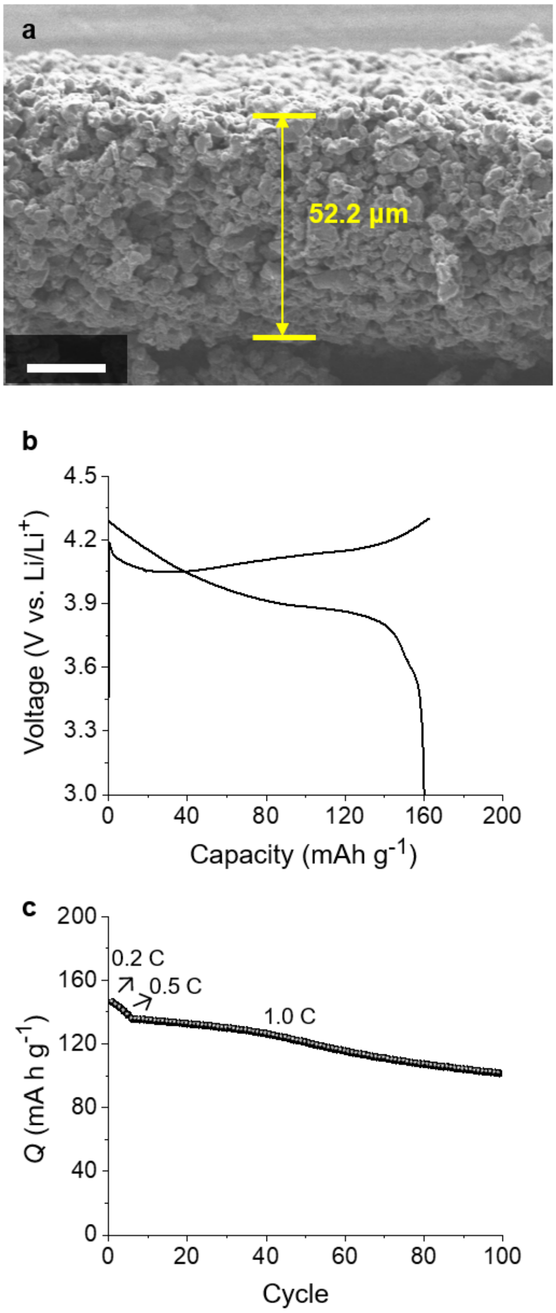

Supplementary Figure 23. Characterization of LCO cathode. **a** Thickness of pristine electrode. **b** Voltage profile of a formation cycle in 3.0-4.3V potential window at 0.1 C-rate. **c** Electrochemical performance of prolonged cycle at marked C-rate in the graph. Scale bar: **a** 20 µm.

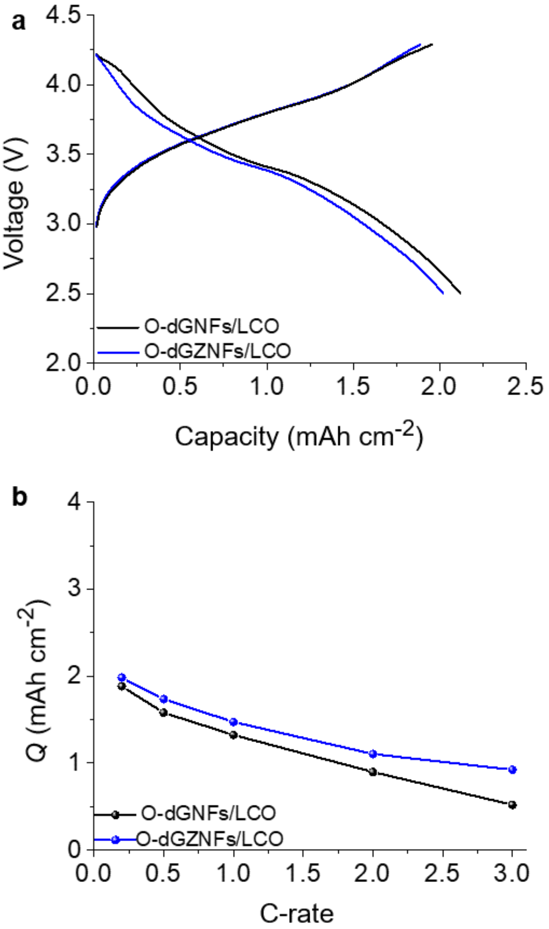

Supplementary Figure 24. Electrochemical properties of O-GNFs/LCO and O-dGZNFs/LCO full cells. **a** Discharge/charge profile of O-GNFs/LCO and O-dGZNFs/LCO in the formation cycle. **b** The plot of capacities depending on various C-rate of full cells paired with LCO cathode in the potential window of 2.5-4.29 V (charge rate = discharge rate).

Supplementary Table 1. Comparison of Li ion diffusion coefficients calculated from **Fig. 3f**.

Sample	Ion diffusion coefficient (cm^{-2} s^{-1})	Sample	Ion diffusion coefficient (cm^{-2} s^{-1})
O-dGNFs-1st	8.958×10^{-8}	O-dGZNFs-1st	2.702×10^{-7}
O-dGNFs-10th	1.092×10^{-7}	O-dGZNFs-10th	2.726×10^{-7}
O-dGNFs-50th	1.257×10^{-7}	O-dGZNFs-50th	3.462×10^{-7}

Supplementary Movies

1. Supplementary Movie 1: First lithiation of O-dGZNF under bias of -2.0 V. The frame speed is 128X times of the real time.
2. Supplementary Movie 2: First delithiation of O-dGZNF under bias of +2.0 V. The frame speed is 96X times of the real time.
3. Supplementary Movie 3: First lithiation of O-dGNF under bias of -2.0 V. The frame speed is 128X times of the real time.
4. Supplementary Movie 4: LED lighting up operation of O-dGZNFs/LCO full-cell. The frame speed is 128X times of the real time.
