# Supplementary material for: Atomic-scale combination of germanium-zinc nanofibers for structural and electrochemical evolution
Source: Nat Commun. 2019 May 30;10:2364. doi: 10.1038/s41467-019-10305-x (PMC6542799; doi:10.1038/s41467-019-10305-x)
Supplement: Supplementary file 3 — Description of Additional Supplementary Files [file 41467_2019_10305_MOESM3_ESM.pdf]

Description of Additional Supplementary Files

Supplementary Movie 1:
First lithiation of O-dGZNF under a bias of -2.0 V. The frame speed is 128X real time.

Supplementary Movie 2:
First delithiation of O-dGZNF under bias of +2.0 V. The frame speed is 96X real time.

Supplementary Movie 3:
First lithiation of O-dGNF under a bias of -2.0 V. The frame speed is 128X real time.

Supplementary Movie 4: LED lighting during operation of the O-dGZNFs/LCO full-cell. The frame speed is 128X real time.
